# A Novel Mechanism of Mesenchymal Stromal Cell-Mediated Protection against Sepsis: Restricting Inflammasome Activation in Macrophages by Increasing Mitophagy and Decreasing Mitochondrial ROS

**DOI:** 10.1155/2018/3537609

**Published:** 2018-02-13

**Authors:** Shuang Li, Hao Wu, Dong Han, Sai Ma, Wensi Fan, Yabin Wang, Ran Zhang, Miaomiao Fan, Yuesheng Huang, Xiaobing Fu, Feng Cao

**Affiliations:** ^1^Department of Cardiology & National Clinical Research Center of Geriatrics Disease, Chinese PLA General Hospital, Beijing 100853, China; ^2^Department of Cardiology, Chengdu Military General Hospital, Chengdu 610083, China; ^3^Department of Toxicology, School of Public Health, Fourth Military Medical University, Xi'an, Shaanxi 710032, China; ^4^Department of Cardiology, Xijing Hospital, Fourth Military Medical University, Xi'an, Shaanxi 710032, China; ^5^Institute of Burn Research, State Key Laboratory of Trauma, Burn and Combined Injury, Southwest Hospital, Third Military Medical University, Chongqing 400038, China

## Abstract

Sepsis, a systemic inflammatory response to infection, is the leading cause of death in the intensive care unit (ICU). Previous studies indicated that mesenchymal stromal cells (MSCs) might have therapeutic potential against sepsis. The current study was designed to investigate the effects of MSCs on sepsis and the underlying mechanisms focusing on inflammasome activation in macrophages. The results demonstrated that the bone marrow-derived mesenchymal stem cells (BMSCs) significantly increased the survival rate and organ function in cecal ligation and puncture (CLP) mice compared with the control-grouped mice. BMSCs significantly restricted NLRP3 inflammasome activation, suppressed the generation of mitochondrial ROS, and decreased caspase-1 and IL-1*β* activation when cocultured with bone marrow-derived macrophages (BMDMs), the effects of which could be abolished by Mito-TEMPO. Furthermore, the expression levels of caspase-1, IL-1*β*, and IL-18 in BMDMs were elevated after treatment with mitophagy inhibitor 3-MA. Thus, BMSCs exert beneficial effects on inhibiting NLRP3 inflammasome activation in macrophages primarily via both enhancing mitophagy and decreasing mitochondrial ROS. These findings suggest that restricting inflammasome activation in macrophages by increasing mitophagy and decreasing mitochondrial ROS might be a crucial mechanism for MSCs to combat sepsis.

## 1. Introduction

Sepsis syndrome, the inflammatory response to infection, is one of the leading causes for death in hospitalized patients [1]. Complications of sepsis, such as acute respiratory distress syndrome (ARDS) and multiple organ dysfunction syndrome (MODS), are major causes for morbidity and mortality in critical patients. Sepsis-associated mortality in the intensive care unit (ICU) is extremely high, with rates of 20% for sepsis, 35% to 45% for severe sepsis, and 60% for septic shock [2]. Therefore, it is essential to identify effective therapeutic strategies for sepsis.

The pathophysiologic process for sepsis is complicated, including invading microorganisms, proinflammatory response, anti-inflammation, and associated immunoparalysis [3, 4]. Extensive data have demonstrated that mesenchymal stromal cells (MSCs), a type of adult stem-like cells, are capable of inhibiting inflammation and immunity responses [5]. Therefore, stem cells have drawn growing interest in the treatment of inflammatory diseases. Of note, it has been reported that MSCs can improve organ function and decrease mortality [6, 7] as well as reduce sepsis-induced inflammation in an animal sepsis model induced by cecal ligation and puncture (CLP) [8]. However, the underlying mechanism is poorly understood, which vastly limits the therapeutic potential of cytotherapy. Previous studies have shown that mitophagy is a prosurvival mechanism associated with cellular exposure to various mitochondrial stressors [9]. Recent studies have indicated that excessive reactive oxygen species (ROS) production is involved in activating mitophagy [10, 11]. It is also well established that the cytosolic E3 ubiquitin ligase, Parkin, and the outer mitochondrial membrane kinase, PTEN-induced putative kinase 1 (PINK1), are two main regulators of mitophagy in mammalian cells [9, 12, 13].

Based on previous reports, we hypothesized that bone marrow-derived mesenchymal stem cells (BMSCs) could reduce sepsis-associated inflammation and organ dysfunction in CLP-induced sepsis. We further investigated the immune-modulatory effects of BMSCs on bone marrow-derived macrophages (BMDMs) to determine the mechanisms for the beneficial effects of BMSCs against sepsis.

## 2. Materials and Methods

### 2.1. Ethics

All animal procedures were conducted in conformity with the National Institutes of Health Guide for the Care and Use of Laboratory Animals. The experimental protocol was approved by the Chinese PLA General Hospital and Fourth Military Medical University Committee on Animal Care.

### 2.2. Animal Study Protocol and CLP Procedures

Transgenic C57BL/6-Tg (CAG-EGFP)1Osb/J mice (C57BL/6, 8–10 weeks, 20–24 g), which continuously express eGFP in all tissues and organs, were commercially purchased from Jackson Laboratory (stock number 003291). Wild-type C57BL/6 mice (8–10 weeks, 20–24 g) were from Animal Laboratory of Fourth Military Medical University. All animals had free access to water and food during the experiment. C57BL/6 mice that underwent the CLP operation were randomly divided into three groups depending on the intravenously injected solution at 1 h postoperation (*n* = 20 in each group), the CLP + saline group (injected with 100 *μ*l of saline); CLP + Fbs group (injected with 2.5 × 10^5^ fibroblasts in 100 *μ*l); and CLP + BMSC group (injected with 2.5 × 10^5^ BMSCs in 100 *μ*l). The fate of BMSCs and myocardial function were detected 24 h after the operation. Visualization of BMSCs in C57BL/6 mice liver tissue at 1–6 h after intravenous injection was shown in supplemental Figure S1C. The survival rate was analyzed every 6 h after the operation until 96 h.

CLP was performed according to the protocol proposed by Rittirsch et al. [14]. Briefly, C57BL/6 mice were anesthetized with persistently inhaled 2% isoflurane during the operation. The abdominal zone was shaved and prepared with 70% ethanol. A ventral midline incision (approximately 1 cm) was made, and the cecum was exteriorized. The cecum was ligated at the designated position with silk suture and penetrated through-and-through with a 22 G needle. Then, the abdominal incision was closed. In the sham group, the cecum was exteriorized, without being ligated or punctured. Immediately after surgery, animals were subcutaneously injected with 1 ml/30 g saline and imipenem-cilastatin for fluid resuscitation and infection prevention.

### 2.3. Cardiac Function Evaluation by Echocardiography

Echocardiography was performed using the Vevo 2100 ultrasound system (Visual-Sonics, Toronto, Canada) with a 30 MHz linear transducer. Anesthesia was conducted with persistently inhaled 1.0% isoflurane. Data of the left ventricular end-diastolic diameter (LVEDd), left ventricular end-systolic diameter (LVESd), and left ventricle (LV) internal dimension in diastole (LVID, d) and systole (LVID, s) were measured. The left ventricular ejection fraction (LVEF) and fractional shortening (FS) were calculated accordingly. The whole procedure was performed by two blinded investigators.

### 2.4. Isolation, Culturing, and Characterization of BMSCs and Preparation of BMDMs

BMSCs^eGFP+^ continuously expressing eGFP were isolated from transgenic C57BL/6-Tg(CAG-EGFP)1Osb/J mice and cultured as previously described [15]. Isolated BMSCs were uniformly negative for CD34, CD45, and Sca-1; were positive for CD29, CD44, and CD90; and had multidifferentiation potential for adipogenesis and osteogenesis (shown in supplemental Figure S1A and B).

Mice were sacrificed, and femurs were removed and cleansed of tissue. Marrows were flushed from the femurs with PBS and collected by centrifugation (200*g*, 5 min). Cells were cultured at the density of 5 × 10^5^/ml in culture dishes with DMEM medium supplemented with 10% (vol/vol) FBS, 1% (vol/vol) penicillin and streptomycin. Following 6 h of incubation at 37°C in 5% CO_2_, nonadherent cells (primary bone marrow-derived macrophages) were decanted and seeded in plates and incubated in complete medium with 25% (vol/vol) conditioned medium from L929 mouse fibroblasts for 7 days (over 90% cells are positive for the cell type surface marker CD11b) to form proliferative nonactivated cells (M0 macrophages) [16, 17].

The *in vitro* cell study was divided into the following six groups: (1) control group (control); (2) LPS-treated group (LPS), BMDMs were primed with LPS (2 *μ*g/ml) for 4 hours; (3) LPS + ATP-treated group (LPS + ATP-stimulated), BMDMs were primed with LPS (2 *μ*g/ml) for 4 hours followed by incubation with ATP (5 mM) for 0.5 h; (4) BMSC coculture group (BMSCs); (5) BMSC coculture and LPS-treated group (BMSC-LPS); and (6) BMSC coculture and LPS + ATP-treated group (BMSC-treated), BMDMs were primed with LPS (2 *μ*g/ml) for 4 hours followed by incubation with ATP (5 mM) for 0.5 h. BMSCs, in transwell coculture, were added to BMDMs in the ATP stimulation step.

### 2.5. Evaluation of Inflammatory Cytokines and Organ Injury Markers

Inflammatory cytokines (IL-1*β*, TNF-*α*, IL-6, and IL-10) and serum biomarkers (cTnI, CK, LDH, ALT, AST, Amylase, Scr, and BUN) were measured with spectrophotometrically commercial ELISA assay kits (R&D Systems, USA) according to the manufacturers' instructions.

### 2.6. Immunohistochemical Staining

Immunohistochemical staining was performed to detect inflammatory cell infiltration. Tissue sections were blocked with 5–10% normal goat serum for 30 min and then incubated with monoclonal antibodies of anti-Ly-6G and anti-Mac-3 (from Biolegend, San Diego, CA, USA) overnight at 4°C. Then, sections were washed and incubated with secondary horseradish peroxidase-conjugated goat anti-rabbit antibodies (from Zhongshan Biotechnology Co. Ltd., Beijing, China) at 37°C for 1 h. Sections were randomly selected, and images were visualized and photographed with inverted or confocal microscopy (Olympus, Japan).

### 2.7. Western Blot

Western blot was performed as previously described [18]. Briefly, proteins were harvested from cells or tissues with RIPA Lysis Buffer (Beyotime Biotechnology, Beijing, China). Total proteins were loaded onto SDS-PAGE gels and transferred electrophoretically to PVDF membranes (Millipore, Billerica, MA). After blocking with 5% skim milk for 1 h, the membranes were incubated with primary antibody at 4°C overnight. Afterwards, membranes were washed and incubated with corresponding secondary antibody at 37°C for 1 h. The blots were developed with enhanced chemiluminescence (ECL) reagent (Millipore) and visualized using UVP Bio-Imaging Systems. Blot densities were analyzed with Quantity One System Software.

Primary antibodies are as follows: procaspase-1, caspase-1, pro-IL-1*β*, P2X7, ASC, NLRP3, PINK1, Parkin, LC3, and *β*-actin (all from Abcam, Cambridge, MA, USA). Secondary antibodies are as follows: horseradish peroxidase-conjugated goat anti-rabbit and goat anti-rat (Zhongshan Biotechnology Co. Ltd.).

### 2.8. Transmission Electron Microscopy (TEM)

TEM was performed to observe the mitochondrial morphology. Briefly, collected BMDMs underwent the procedures of fixation, stepwise alcohol dehydration, embedding, polymerization, sectioning, and staining. Images were observed with an electron microscope (JEM-2000EX TEM, JEOL Ltd., Tokyo, Japan). Random sections were visualized by a blinded technician.

### 2.9. Mitochondrial ROS Production Detection

Mitochondrial ROS production was detected using MitoSOX™ (Ex/Em: 510/580 nm), MitoTracker® Deep Red FM (Ex/Em: 640/662 nm), and MitoTracker Green FM (Ex/Em: 490/516 nm) fluorescent dye (all from Invitrogen, Invitrogen Corporation, USA) by flow cytometry.

### 2.10. Statistical Analysis

Data were expressed as the mean ± SD and were analyzed using ANOVA followed by a Bonferroni correction for the post hoc *t*-test. The survival rate was analyzed with the Kaplan-Meier test followed by the log-rank post hoc test. All statistical tests were performed using SPSS software version 17.0 (IBM, Armonk, NY, USA) and GraphPad Prism software version 5.0 (GraphPad Software, San Diego, CA). A value of *p* < 0.05 was considered statistically significant.

## 3. Results

### 3.1. BMSC Treatment Improved Survival Rate and Multiorgan Functions after CLP

**Figure 1:**
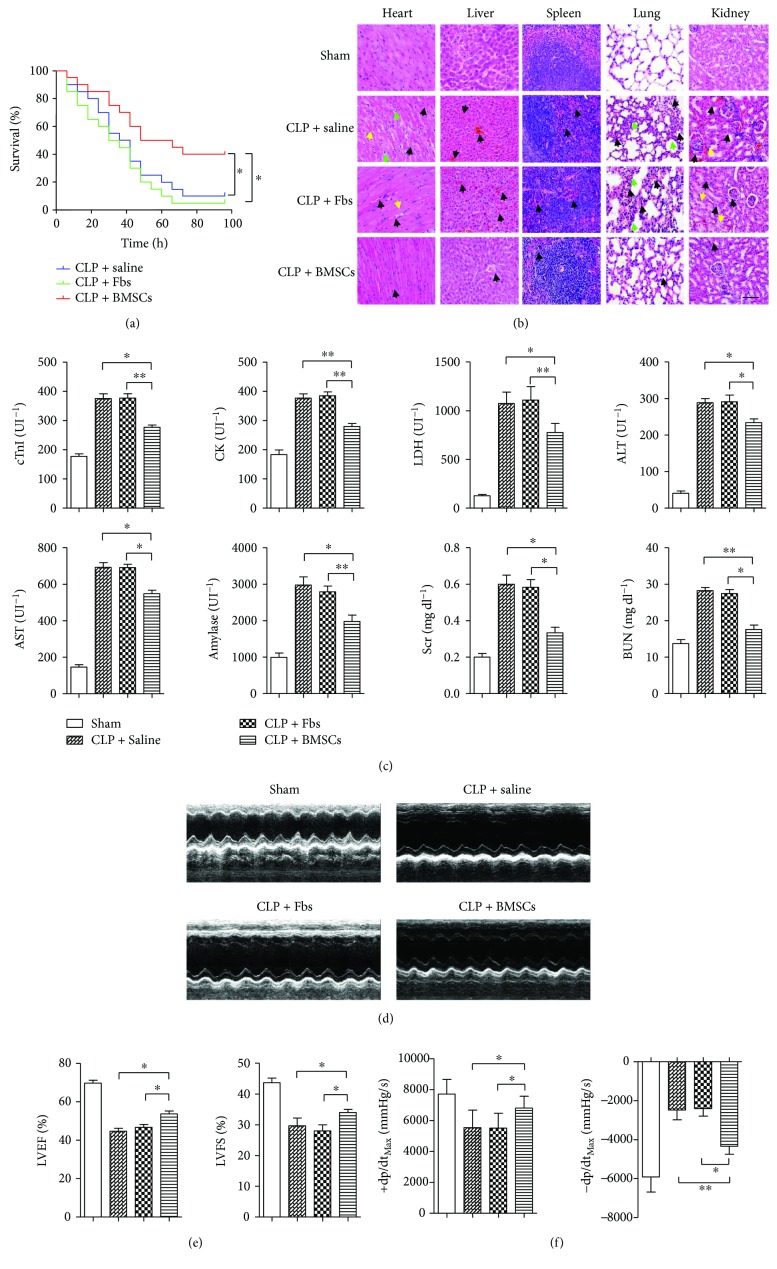
Effect of intravenous injection of BMSCs on the course of sepsis after CLP. (a) Survival curves of C57BL/6 mice after CLP and treatment using BMSCs as well as fibroblasts. (b) Representative images of organs from mice that received saline or BMSCs after CLP surgery. The arrows point to interstitial edema (yellow arrows), red blood cell infiltration (black arrows), and inflammatory cell infiltration (green arrows). Scale bar, 200 *μ*m. (c) Effect of BMSC treatments on CLP-induced multiorgan injury. Serum biomarkers, including cardiac troponin I (cTnI), creatinine kinase (CK), lactate dehydrogenase (LDH), alanine aminotransferase (ALT), aspartate aminotransferase (AST), amylase, serum creatinine values (SCr), and blood urea nitrogen (BUN), were measured in serum by ELISA. (d) Representative M-mode echocardiography images of mice. (e) Measurement of the left ventricular ejection fraction (LVEF) and left ventricular fractional shortening (LVFS). (f) Measurement of the maximal velocity increase and decrease in the pressure per second in the left ventricle (±dP/dt). Error bars represent the means ± s.e.m. *n* = 10–15 per group. ^∗^ *p* < 0.05 and ^∗∗^ *p* < 0.01. The data are representative of three independent experiments.

Figure 1(a) shows the survival curve of C57BL/6 mice after CLP. Compared with the CLP + saline and CLP + Fbs groups, the survival rate was significantly increased in the CLP + BMSC group, with survival rates of 10%, 5%, and 40% in the three groups at the time point of 96 h (*p* < 0.05). As revealed by HE staining, BMSC observably improved morphological changes in vital organs, such as the heart, liver, spleen, lungs, and kidneys, which was manifested by interstitial edema, red blood cell, and inflammatory cell infiltration (Figure 1(b)). In addition, serum biomarkers, including cardiac troponin I (cTnI), creatinine kinase (CK), lactate dehydrogenase (LDH), alanine aminotransferase (ALT), aspartate aminotransferase (AST), amylase, serum creatinine values (SCr), and blood urea nitrogen (BUN), were significantly reduced in the CLP + BMSC group compared with the CLP + Saline and CLP + Fbs groups (*P* < 0.05), indicating that BMSC treatment improved organ functions after CLP (Figure 1(c)).

### 3.2. BMSCs Improved Myocardial Function after CLP

Figure 1(d) shows representative M-mode echocardiography images in each group. CLP-induced cardiac depression was manifested by a decreased left ventricular ejection fraction (LVEF), left ventricular fractional shortening (LVFS), and maximal velocity increase as well as a decrease in the pressure per second in the left ventricular (±dP/dt) in the CLP + saline group (*p* < 0.05, CLP + saline group versus sham group). In comparison with the CLP + saline and CLP + Fbs group, BMSCs had an increased left ventricular ejection fraction (LVEF), left ventricular fractional shortening (LVFS), and maximal velocity as well as a decrease in pressure per second in the left ventricle (±dP/dt), suggesting that BMSC treatment improved myocardial function after CLP (Figures 1(e) and 1(f)).

### 3.3. BMSCs Reduced the CLP-Induced Inflammation Level

**Figure 2:**
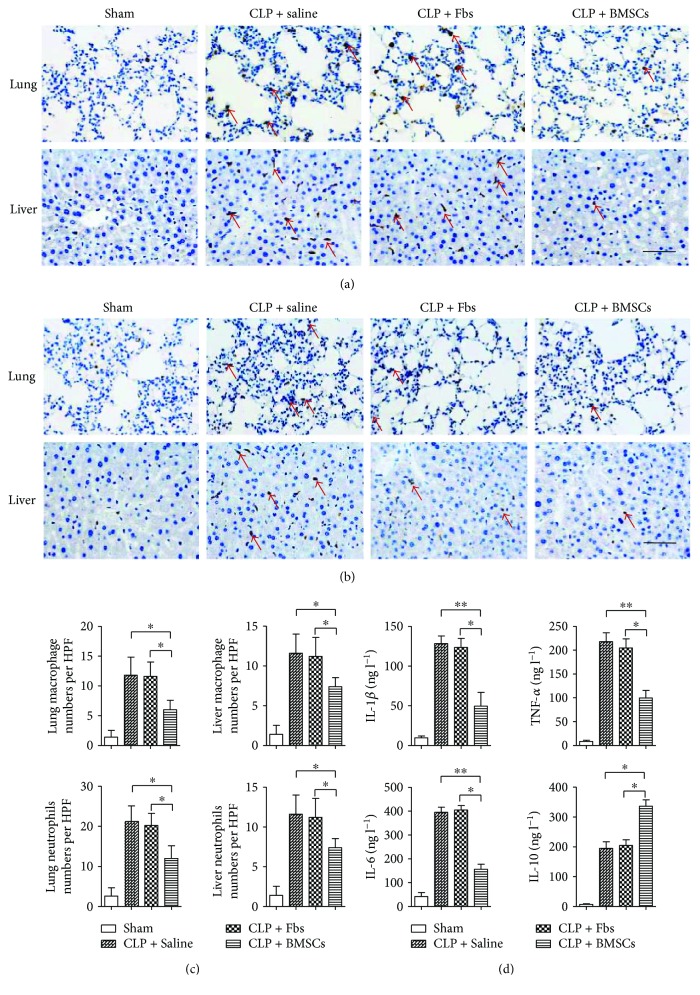
Treatment with BMSCs reduced the levels of sepsis-induced inflammation. (a-b) Treatment with BMSCs attenuated sepsis-induced inflammatory cell infiltration in organs. Inflammatory cell infiltration was determined by immunohistochemistry staining. Red arrows indicate inflammatory cells. (a) The sections were immunohistochemically analyzed using antibodies against Mac-3 for macrophages (red arrows). Scale bar, 100 *μ*m. (b) The sections were immunohistochemically analyzed using antibodies against Ly6G for neutrophils (red arrows). Scale bar, 100 *μ*m. (c) Quantitative analysis of positive cell numbers per five different HPFs (high magnification fields). (d) Levels of the proinflammatory cytokines IL-6, IL-1*β*, and TNF-*α* and anti-inflammatory cytokine IL-10 in serum were measured by ELISA. Error bars represent the means ± s.e.m. *n* = 10–15 per group. ^∗^ *p* < 0.05 and ^∗∗^ *p* < 0.01. Data are representative of three independent experiments.

Inflammatory cell infiltration in the heart and lung tissue was determined by the immunohistochemistry for Ly-6G and MAC-3, which are well-recognized biomarkers for neutrophils and macrophages. It could be inferred that BMSC treatment inhibited inflammatory cell infiltration, as evidenced by decreased macrophage (Mac-3) (Figures 2(a) and 2(c)) and neutrophil (Ly6G) infiltration (Figures 2(b) and 2(c)) in the lung and liver tissues. Furthermore, BMSCs decreased the proinflammatory cytokine levels of IL-6, IL-1*β*, and TNF-*α*, while increasing anti-inflammatory cytokine IL-10 in the serum (Figure 2(d), *p* < 0.05, CLP + BMSC group versus CLP + saline group; *p* < 0.05, CLP + BMSC group versus CLP + Fbs group).

### 3.4. BMSCs Inhibited NLRP3 Inflammasome-Mediated Caspase-1 and IL-1*β* Activation

**Figure 3:**
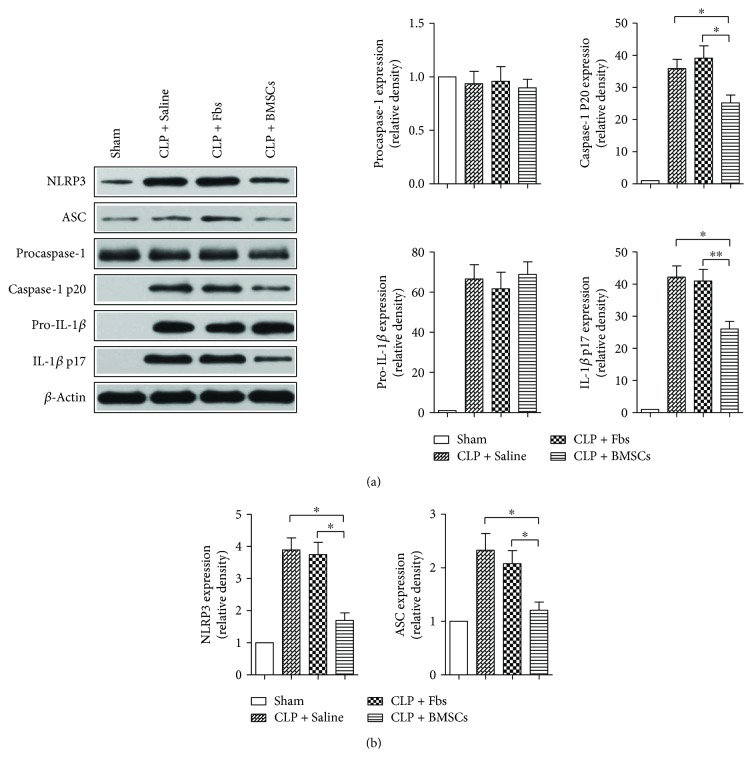
BMSC treatment inhibited NLRP3 inflammasome-mediated and IL-1*β* activation in liver tissues of C57BL/6 mice. (a) Representative blots of P2X7, NLRP3, ASC, procaspase-1, pro-IL-1*β*, caspase-1, and IL-1*β* in livers subjected to CLP. (b) Semiquantitative analysis of the Western blots. ^∗^ *p* < 0.05 and ^∗∗^ *p* < 0.01. *n* = 5–10. Data are representative of three or more independent experiments.

Figure 3(a) shows representative blots of NLRP3, ASC, procaspase-1, caspase-1 p20, pro-IL-1*β*, and IL-1*β* in liver tissue. NLRP3 inflammasome expression was decreased after BMSC treatment as compared with the CLP + saline and CLP + Fbs groups (Figures 3(a) and 3(b)). Moreover, caspase-1 cleavage and activation, leading to maturation and secretion of IL-1*β*, revealed by caspase-1 p20 and IL-1*β* p17 expression, were more suppressed in liver tissues of mice with BMSC treatment than that with single saline or Fbs treatment.

### 3.5. BMSCs Inhibited Caspase-1 and IL-1*β* Activation in BMDMs

**Figure 4:**
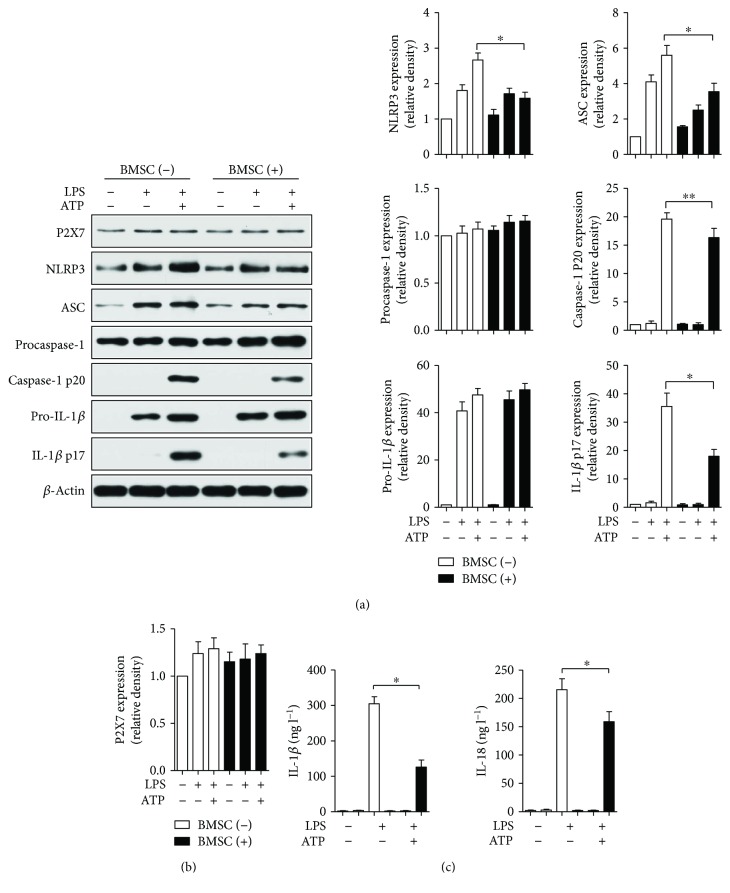
BMSCs inhibited NLRP3 inflammasome-mediated caspase-1 activation and IL-1*β* and IL-18 secretion in bone marrow-derived macrophages (BMDMs). BMDMs were primed with LPS (2 *μ*g/ml) for 4 hours, which was followed by incubation with ATP (5 mM) for 0.5 h. BMSCs, in transwell coculture, were added to BMDMs at the ATP stimulation step. At 18 hours, caspase-1 and IL-1*β* activation was analyzed in lysates of macrophages by Western blot (a, b), and IL-1*β* and IL-18 secretion in the supernatants was quantified by ELISA (c). Error bars represent the means ± s.e.m. ^∗^ *p* < 0.05 and ^∗∗^ *p* < 0.01. *n* = 5–10. The data are representative of three or more independent experiments.

Briefly, BMDMs were primed with LPS (2 *μ*g/ml) for 4 hours, which was followed by incubation with ATP (5 mM) for 0.5 h. BMSCs, in transwell coculture, were added to BMDMs at the ATP stimulation step. Then, 18 hours later, caspase-1 and IL-1*β* activation were analyzed in macrophage lysates by Western blot (shown in Figure 4(a)). Both LPS and ATP stimulation induced caspase-1 and IL-1*β* activation, as evidenced by increased caspase-1 p20 and IL-1*β* p17 expression. Furthermore, BMSC coculturing suppressed LPS and ATP stimulation induced caspase-1 and IL-1*β* activation in BMDMs (*p* < 0.01, *p* < 0.05, BMSC-treated group versus LPS + ATP-stimulated group) (Figure 4(b)). Similarly, the levels of IL-1*β* and IL-18 in the supernatants were reduced in the BMSC-treated group compared with the LPS + ATP-stimulated group (Figure 4(c)). In addition, there was no significant difference in the expression levels of P2X7, procaspase-1, and pro-IL-1*β* among the groups (Figure 4(b)).

### 3.6. BMSCs Inhibited NLRP3 Inflammasome Activation in BMDMs by Decreasing Mitochondrial ROS

**Figure 5:**
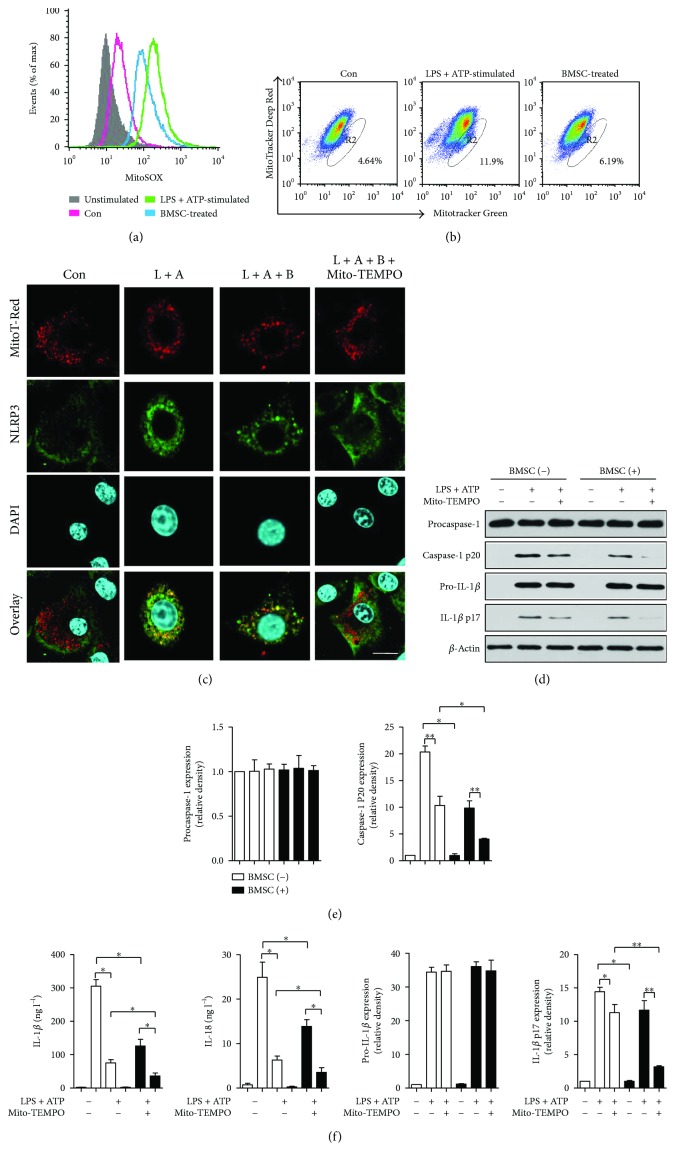
BMSCs negatively regulate the NLRP3 inflammasome in BMDMs by decreasing mitochondrial ROS (mt ROS). (a), (b) BMDMs were treated with LPS (2 *μ*g/ml, 4 hours) and ATP (5 mM, 0.5 h) (L + A). BMSCs were cocultured with BMDMs in transwell from ATP licensing for 2 h (L + A + B). BMDMs were stained with MitoSOX (5 *μ*M) (a) or MitoTracker Deep Red (500 nM) and MitoTracker Green (200 nM) (c) for the final 30 min and then analyzed by flow cytometry. (c) Colocalization of the NLRP3 and mitochondria. BMDMs expressing NLRP3 (green) were analyzed for the colocalization of NLRP3 with the mitochondria (red) using confocal microscopy. Scale bar, 20 *μ*m. (d–f) Inhibition of mtROS generation abolished caspase-1 activation. Western blot analysis for caspase-1 and IL-1*β* in lysates (d, e) and cytokine secretion (f) of BMDMs incubated for 1 h with Mito-TEMPO (500 *μ*M), which was followed by LPS and ATP. Error bars represent the means ± s.e.m. ^∗^ *p* < 0.05 and ^∗∗^ *p* < 0.01. *n* = 5–10. The data are representative of three or more independent experiments.

Compared with the LPS + ATP-stimulated group, BMSC coculturing reduced the mitochondrial ROS (mtROS) level in BMDMs (Figure 5(a)). The flow cytometry analysis of MitoTracker Deep Red-MitoTracker Green (Figure 5(b)) indicated that BMSCs alleviated LPS + ATP stimulation-induced mitochondrial damage in BMDMs. Antioxidant Mito-TEMPO was used to inhibit mtROS generation.  Figure 5(c) shows the colocalization of NLRP3 (green), mitochondria (red), and nucleus (blue) by immunofluorescence staining. In control BMDMs, very little NLRP3 cosedimented with mitochondria. However, much more NLRP3 was colocalized with or adjacent to mitochondria after LPS-ATP stimulation. Both BMSCs and Mito-TEMPO resulted in less colocalization of NLRP3 and mitochondria. After LPS and ATP stimulation, Mito-TEMPO treatment decreased the expression levels of caspase-1 p20 and IL-1*β* p17 (*p* < 0.01, *p* < 0.05), suggesting that NLRP3 activation was associated with mtROS level. Additionally, the expression levels of caspase-1 p20 and IL-1*β* p17 were also reduced in the BMSC-treated group (*p* < 0.01, *p* < 0.01), indicating that the effect of BMSCs on BMDMs could be attributed to mtROS scavenge (Figures 5(d) and 5(e)). Moreover, both BMSCs and Mito-TEMPO decreased the cytokine secretions of IL-1*β* and IL-18 (Figure 5(f), *P* < 0.05).

### 3.7. BMSCs Inhibited NLRP3 Inflammasome Activation by Increasing BMDM Mitophagy

**Figure 6:**
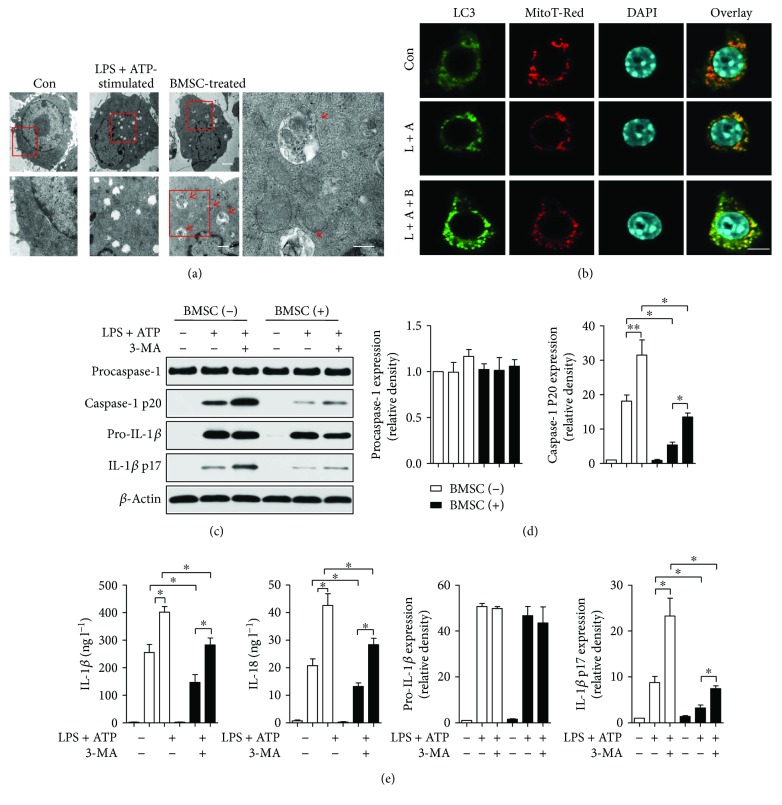
BMSCs inhibited NLRP3 inflammasome activation in BMDMs by increasing the mitophagy of BMDMs. (a) Electron microscopy images of BMDMs showing mitochondrial morphologic changes. Red arrows indicate mitophagy. Scale bar, 2 *μ*m (top row), 600 nm (bottom row, left), and 400 nm (bottom row, right). (b) Confocal microscopy analysis of BMDMs stained for LC3 (green) and mitochondria (red) for colocalization as an indicator of mitophagy. Scale bar, 20 *μ*m. (c–e) Inhibition of mitophagy results in caspase-1 activation. Western blot analysis for caspase-1 and IL-1*β* in lysates (c, d) and cytokine secretion (e) of BMDMs incubated for 6 h with 3-methyladenine (3-MA) (10 mM), which was followed by LPS and ATP. Error bars represent the means ± s.e.m. ^∗^ *p* < 0.05 and ^∗∗^ *p* < 0.01. *n* = 5–10. The data are representative of three or more independent experiments.

Using electron microscopy (EM), we directly assessed the mitochondrial integrity and state. We observed more mitophagy (indicated by red arrows) and fewer swollen mitochondria in the BMSC-treated group than for treatment with LPS and ATP in BMDMs (Figure 6(a)). In addition, autophagy-associated LC3 puncta were found to accumulate around the mitochondria in BMDMs when coculturing with BMSCs after stimulation by LPS and ATP (Figure 6(b)). As was shown by the Western blot results in Figures 6(c) and 6(d), treatment of BMDMs with the mitophagy/autophagy inhibitor 3-methyladenine (3-MA) enhanced the activation of caspase-1 and IL-1*β*, as well as the secretion of IL-1*β* and IL-18 in BMDMs when coculturing with BMSCs after LPS and ATP stimulation (Figure 6(e)).

### 3.8. BMSCs Increased Parkin-Mediated Mitophagy in BMDMs

**Figure 7:**
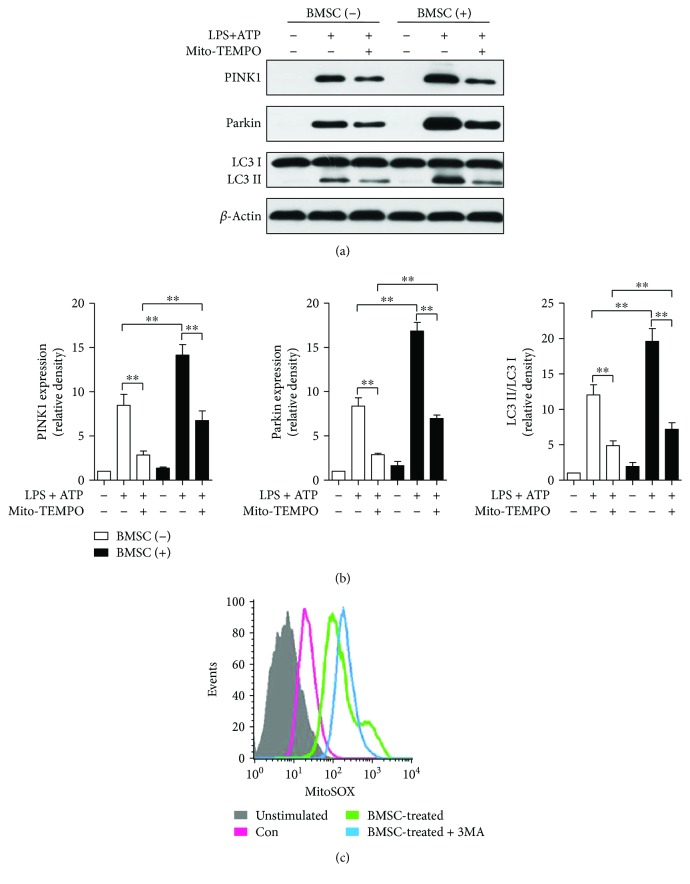
BMSCs increased Parkin-mediated mitophagy and decreased mtROS generation of BMDMs. (a-b) Western blot analysis for caspase-1 and IL-1*β* in lysates (a, b) of BMDMs incubated for 1 h with Mito-TEMPO (500 *μ*M), which was followed by LPS and ATP. (c) BMDMs stimulated with 3-MA (10 mM) for 6 h were stained with MitoSOX (5 *μ*M) for 30 min and were then analyzed by flow cytometry. Error bars represent the means ± s.e.m. ^∗∗^ *p* < 0.01. *n* = 5–10. The data are representative of three or more independent experiments.

Both BMSCs and Mito-TEMPO significantly elevated the expression of PINK1, Parkin, and LC3BII/I ratio (Figures 7(a) and 7(b)). MitoSOX flow cytometry revealed that BMSCs significantly decreased mtROS generation, while mitophagy inhibition by 3-MA diminished this effect, indicating that the inhibitory effects of BMSCs on mtROS generation were mediated by mitophagy (Figure 7(c)).

## 4. Discussion

Several studies have indicated the beneficial effects of MSCs against sepsis, but the underlying mechanisms remain unclear. In the present study, we demonstrate that BMSCs exert beneficial effects on experimental sepsis *via* inhibiting NLRP3 inflammasome activation in macrophages. Furthermore, one important finding in the present study was that BMSCs restricted NLRP3 inflammasome activation through increasing mitophagy activation and decreasing mitochondrial ROS in macrophages.

The antisepsis efficacy of MSCs may be attributed to their ability to home injured tissue, secrete paracrine cytokines, decrease apoptosis in injured tissues, and modulate immune cells [19, 20]. It was reported that MSCs attenuated septic injury by reducing the infiltration of inflammatory cells and cell death in various targeted organs [21]. However, Krasnodembskaya et al. determined that human BMSCs attenuated live bacteria-induced injury due to direct antimicrobial activity [22]. Moreover, the same group has published a study on therapeutic potential of human MSC in the model of sepsis, in which they found that MSC effect was associated with increased phagocytic activity of blood monocytes and M2 polarization of monocytes/macrophages in the spleen [23]. Although complex, the acquisition of a mechanistic knowledge is essential for developing BMSC cell therapy against sepsis injury. In the present study, our data showed that BMSCs increased the CLP murine survival rate and alleviated organ injury via improving organ function and alleviating proinflammatory cytokines.

Cardiac function is crucial to the clinical outcomes of sepsis. Therefore, myocardial dysfunction has been gaining increasing attention in recent years [24, 25]. In the current study, we observed impaired cardiac function in septic mice by echocardiography and hemodynamic measurements. BMSC treatment effectively improved cardiac function induced by CLP. Similarly, the results of Weil et al. suggested that intravenous infusion of MSCs improved myocardial function in the endotoxemia model [26]. However, the precise mechanism of MSCs' beneficial function in cardiac recovery is far from clear. Data from Rogers et al. revealed that MSCs exerted reparative effects on myocardium through molecular reprogramming of the cardiomyocytes themselves [27]. Notably, studies on sepsis indicate that mitochondria could mediate organ dysfunction, including heart function depression. Considering our data, mitochondrial dysfunction may be a fundamental contributor to cardiac depression in sepsis. However, further studies are still needed to clarify this.

According to previous studies, sepsis is initiated when well-conserved microbial structures (also known as pathogen-associated molecular patterns, PAMPs) bind to receptors embedded either on the cell membranes or inside the cell cytoplasm of cells of the innate immune system, namely, blood monocytes and tissue macrophages. The characteristic of the first phase of sepsis is the production of proinflammatory cytokines, including tumor necrosis factor-alpha (TNF-*α*), interleukin- (IL-) 1*β*, IL-6, and IL-8. These proinflammatory mediators orchestrate septic reaction of the host. Soon after this first phase of hyperproduction of proinflammatory mediators, a second phase ensues during which Th2 cells, monocytes, and macrophages stimulated by PAMPs secrete a large amount of anti-inflammatory mediators like IL-10. This phase is considered a state of immunosuppression or immunoparalysis of the host when multiple organ dysfunctions take place [28]. Consistent with previous reports, we found that the inflammatory biomarkers (IL-1*β*, TNF-*α*, IL-6, and IL-10), serum markers (cTnI, CK, LDH, ALT, AST, Amylase, Scr, and BUN), and inflammatory cell infiltration were increased in CLP mice compared with those in control mice [7, 26, 29]. In addition, mitochondrial ROS generation was also elevated in CLP-induced septic mice. These results were consistent with the findings by Chang et al., who proposed that CLP-induced sepsis triggered a rigorous inflammatory response with the generation of ROS [30]. Sepsis is characterized by overwhelming activation of inflammatory and immune responses. An interesting finding in the present study was that BMSCs restricted the inflammatory responses and ROS generation in CLP animals. This is comparable to previous reports. Yip and his colleagues reported that combined therapy with melatonin and apoptotic adipose-derived mesenchymal stem cells alleviated sepsis-induced organ injury through attenuating inflammatory reactions and oxidative stress [2, 31]. Notably, the inflammatory cytokines were produced by macrophages rather than injected BMSCs, suggesting that the therapeutic effect of BMSCs against excessive inflammatory responses was mediated by an interaction between BMSCs and macrophages. The *in vitro* coculture experiments further confirmed this finding.

Inflammasomes are a variety of protein complexes that could recognize inflammation-inducing stimuli and control inflammatory cytokine production [32]. Among them, the NLRP3 (nucleotide-binding domain, leucine-rich-repeat-containing family, and pyrin domain-containing 3) inflammasome is one of the most widely studied inflammasomes. The NLRP3 inflammasome, consisting of the regulatory subunit NLRP3, adaptor ASC, and effector caspase-1, is a molecular complex that could be activated to trigger immune defenses through secreting inflammatory cytokines such as IL-1*β* or IL-18 [33, 34]. Previous researches showed that the NLRP3 inflammasome was upregulated and activated in the liver during sepsis [35, 36]. Our data revealed that NLRP3 inflammasome-mediated and IL-1*β* activation in septic liver tissue, and BMSCs inhibited NLRP3 activation and resultant IL-1*β* activation. Although the level of NLRP3 or its adaptor ASC did not reveal a significant alteration, its cellular localization changed. As inferred by our immunofluorescence results, the resting NLRP3 was localized in the endoplasmic reticulum, while under the condition of inflammasome activation, NLRP3 colocalized with endoplasmic reticulum and mitochondrion structures. Mitochondrial ROS was capable of inducing NLRP3 inflammasome activation [37]. As revealed in our data, with the addition of Mito-TEMPO (a mitochondria-targeted antioxidant), the relocalization of NLRP3 inflammasome was further attenuated, in combination with subsequent decreased activation of caspase-1 and IL-1*β*. Similar to the previous results, we observed that N-acetylcysteine (NAC, a general reactive oxygen inhibitor) abrogated the apparent increase in caspase-1 activation and IL-1*β* expression in BMDMs in response to LPS and ATP. These results suggested that the protective effect of BMSCs on the NLRP3 inflammasome was mediated by regulating mitochondrial ROS generation.

Maintaining a healthy mitochondrion population is important for cells. Autophagic clearance is the major pathway in mitochondrial turnover, termed as mitophagy [38]. Recently, Mahrouf-Yorgov et al. found that MSCs that engrafted into infarcted hearts of mice could reduce damage via upregulating HO-1 and increasing mitochondrial biogenesis, and inhibition of mitophagy or HO-1 failed to protect against cardiac apoptosis [39]. Activation of the autophagy process has been reported to attenuate cardiac dysfunction and liver injury in septic mice [40–42]. Similarly, Carchman et al. demonstrated that mitophagy was necessary to prevent organ injury in sepsis, but the authors attributed this protective process to the activation of TLR9 signaling [43]. Parkin, an E3 ubiquitin ligase, promotes dysfunctional mitochondrion clearance by autophagy [44]. In our current study, the results indicated that BMSCs increased Parkin-mediated mitophagy and decreased mtROS generation of BMDMs. Furthermore, inhibition of mitophagy with 3-MA agent blocked the inhibitory role of BMSCs in IL-1*β* activation and mitochondrial ROS production, indicating that the effect of BMSCs on inflammatory regulation and mitochondrial ROS generation was mainly mediated by mitophagy.

Although the data from our study bear some clinical relevance, several limitations remain. First, cardiac function depression in sepsis may be detoxified by various factors, such as inflammatory factors, which may not represent all contributors. We only observed the effects of MSCs, but the underlying mechanisms are still unclear. Second, the data were based on a murine CLP model, and the dose of BMSCs may be different in a clinical setting. Besides, as the *in vitro* cell study cannot fully simulate the *in vivo* circumstances, the weak link between *in vivo* and *in vitro* studies should be taken into consideration. Although the mechanisms of inflammasome inhibition by MSCs were dissected *in vitro*, it was less well confirmed *in vivo*. Therefore, more studies are warranted to confirm if our current findings are true for human MSCs in clinical settings.

## 5. Conclusion

**Figure 8:**
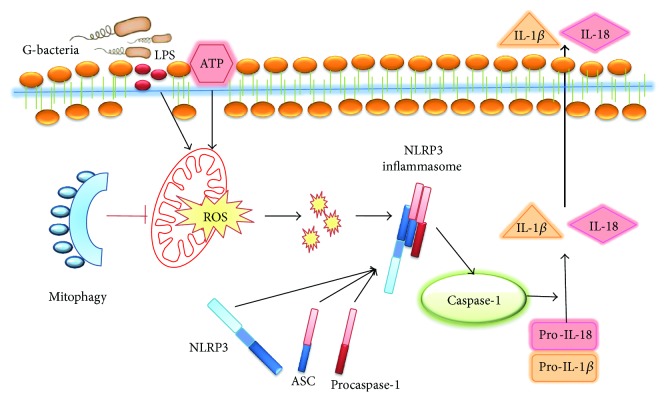
Proposed scheme for the mechanism of mesenchymal stromal cell-mediated protection against sepsis. A summary of our current hypothesis about the mechanisms that underlie the interactions between BMSCs and BMDMs in the CLP sepsis model. BMSCs negatively regulate the NLRP3 inflammasome in BMDMs primarily by increasing mitophagy of BMDMs in response to crosstalk with activated BMDMs and thus by decreasing mitochondrial ROS.

BMSCs improved the murine survival rate and alleviated organ injury in experimental sepsis induced by CLP. BMSCs' beneficial effects were mediated by inhibiting NLRP3 inflammasome activation in macrophages, which was primarily through increasing the mitophagy of macrophages and decreasing mitochondrial ROS generation (Figure 8). Taken together, MSCs can act as promising cellular therapy, as an immune-modulator, for fighting against sepsis.

## Supplementary Materials

Supplementary 1 Figure S1: characterization and fate of injected of BMSCseGFP+. A. Differentiation potential of BMSCseGFP+. Fibroblast-like-shaped BMSCseGFP+ were GFP positive. Adipogenesis of BMSCseGFP+ under the adipogenic differentiation conditions was detected by oil red O staining. Osteogenesis was evaluated by Alizarin red S staining. Scale bar, 100 *μ*m. B. Flow cytometry results show that BMSCseGFP+ were uniformly negative for CD34, CD45, and Sca-1 and positive for CD29, CD44, and CD90. C. Visualization of BMSCseGFP+ (red arrows) in C57BL/6 mice liver tissue at 1–6 h after intravenous injection. Scale bar, 100 *μ*m. Data shown are representative of two (A, B) or three (C) independent experiments.

Supplementary 2 Figure S2: inhibition of ROS generation abolished caspase-1 activation. A. Western blot analysis for caspase-1 and IL-1*β* in lysates of BMDMs incubated for 1 h with NAC (Sigma-Aldrich, USA) (10 mM), which was followed by LPS and ATP. B. Semiquantitative analysis of the Western blots. Error bars represent the means ± s.e.m. ^∗^ *p* < 0.05 and ^∗∗^ *p* < 0.01. *n* = 5–10. The data are representative of at least three independent experiments.

Supplementary 3 Figure S3: confocal microscopy analysis of BMDMs stained for LC3 (green) and mitochondria (red) for colocalization as an indicator of mitophagy. Scale bar, 20 *μ*m.

Supplementary 4 Figure S4: original images of caspase-1 p20 and IL-1*β* p17 in Figure 4.

Supplementary 5 Figure S5: relative mean fluorescence intensity (MFI) comparisons. A. BMDMs were treated with LPS (2 *μ*g/ml, 4 hours) and ATP (5 mM, 0.5 h) (L + A). BMSCs were cocultured with BMDMs in transwell from ATP licensing for 2 h. BMDMs were stained with MitoSOX (5 *μ*M) for the final 30 min and then analyzed by FlowJo 7.6.1(Becton, Dickinson & Company, USA). B. BMDMs stimulated with 3-MA (10 mM) for 6 h were stained with MitoSOX (5 *μ*M) for 30 min and were then analyzed by FlowJo 7.6.1. The data are representative of three independent experiments.

Supplementary 6 Figure S6: colocalization of the NLRP3 and mitochondria. BMDMs expressing NLRP3 (green) were analyzed for the colocalization of NLRP3 with the mitochondria (red) using confocal microscopy. Scale bar, 20 *μ*m.

## References

[1] Liu V., Escobar G. J., Greene J. D. (2014). Hospital deaths in patients with sepsis from 2 independent cohorts. *JAMA*.

[2] Chen H. H., Lin K. C., Wallace C. G. (2014). Additional benefit of combined therapy with melatonin and apoptotic adipose-derived mesenchymal stem cell against sepsis-induced kidney injury. *Journal of Pineal Research*.

[3] Leentjens J., Kox M., van der Hoeven J. G., Netea M. G., Pickkers P. (2013). Immunotherapy for the adjunctive treatment of sepsis: from immunosuppression to immunostimulation. Time for a paradigm change?. *American Journal of Respiratory and Critical Care Medicine*.

[4] Fry D. E. (2012). Sepsis, systemic inflammatory response, and multiple organ dysfunction: the mystery continues. *The American Surgeon*.

[5] Le Blanc K., Tammik L., Sundberg B., Haynesworth S. E., Ringden O. (2003). Mesenchymal stem cells inhibit and stimulate mixed lymphocyte cultures and mitogenic responses independently of the major histocompatibility complex. *Scandinavian Journal of Immunology*.

[6] Gotts J. E., Matthay M. A. (2011). Mesenchymal stem cells and acute lung injury. *Critical Care Clinics*.

[7] Németh K., Leelahavanichkul A., Yuen P. S. T. (2009). Bone marrow stromal cells attenuate sepsis via prostaglandin E_2_-dependent reprogramming of host macrophages to increase their interleukin-10 production. *Nature Medicine*.

[8] Mei S. H. J., Haitsma J. J., Dos Santos C. C. (2010). Mesenchymal stem cells reduce inflammation while enhancing bacterial clearance and improving survival in sepsis. *American Journal of Respiratory and Critical Care Medicine*.

[9] Eid N., Ito Y., Otsuki Y. (2016). Triggering of Parkin mitochondrial translocation in mitophagy: implications for liver diseases. *Frontiers in Pharmacology*.

[10] Zhong Z., Umemura A., Sanchez-Lopez E. (2016). NF-*κ*B restricts inflammasome activation via elimination of damaged mitochondria. *Cell*.

[11] Wang Y., Nartiss Y., Steipe B., McQuibban G. A., Kim P. K. (2012). ROS-induced mitochondrial depolarization initiates PARK2/PARKIN-dependent mitochondrial degradation by autophagy. *Autophagy*.

[12] Geisler S., Holmstrom K. M., Skujat D. (2010). PINK1/Parkin-mediated mitophagy is dependent on VDAC1 and p62/SQSTM1. *Nature Cell Biology*.

[13] Vasquez-Trincado C., Garcia-Carvajal I., Pennanen C. (2016). Mitochondrial dynamics, mitophagy and cardiovascular disease. *The Journal of Physiology*.

[14] Rittirsch D., Huber-Lang M. S., Flierl M. A., Ward P. A. (2009). Immunodesign of experimental sepsis by cecal ligation and puncture. *Nature Protocols*.

[15] Pan Q., Qin X., Ma S. (2014). Myocardial protective effect of extracellular superoxide dismutase gene modified bone marrow mesenchymal stromal cells on infarcted mice hearts. *Theranostics*.

[16] Nakahira K., Haspel J. A., Rathinam V. A. K. (2011). Autophagy proteins regulate innate immune responses by inhibiting the release of mitochondrial DNA mediated by the NALP3 inflammasome. *Nature Immunology*.

[17] Pineda-Torra I., Gage M., de Juan A., Pello O. M. (2015). Isolation, culture, and polarization of murine bone marrow-derived and peritoneal macrophages. *Methods in Molecular Biology*.

[18] Wang D., Luo P., Wang Y. (2013). Glucagon-like peptide-1 protects against cardiac microvascular injury in diabetes via a cAMP/PKA/Rho-dependent mechanism. *Diabetes*.

[19] Wannemuehler T. J., Manukyan M. C., Brewster B. D. (2012). Advances in mesenchymal stem cell research in sepsis. *Journal of Surgical Research*.

[20] Walter J., Ware L. B., Matthay M. A. (2014). Mesenchymal stem cells: mechanisms of potential therapeutic benefit in ARDS and sepsis. *The Lancet Respiratory Medicine*.

[21] Yagi H., Soto-Gutierrez A., Kitagawa Y., Tilles A. W., Tompkins R. G., Yarmush M. L. (2010). Bone marrow mesenchymal stromal cells attenuate organ injury induced by LPS and burn. *Cell Transplantation*.

[22] Krasnodembskaya A., Song Y., Fang X. (2010). Antibacterial effect of human mesenchymal stem cells is mediated in part from secretion of the antimicrobial peptide LL-37. *Stem Cells*.

[23] Krasnodembskaya A., Samarani G., Song Y. (2012). Human mesenchymal stem cells reduce mortality and bacteremia in gram-negative sepsis in mice in part by enhancing the phagocytic activity of blood monocytes. *American Journal of Physiology Lung Cellular and Molecular Physiology*.

[24] Fenton K. E., Parker M. M. (2016). Cardiac function and dysfunction in sepsis. *Clinics in Chest Medicine*.

[25] Latini R., Caironi P., Masson S. (2016). Cardiac dysfunction and circulating cardiac markers during sepsis. *Minerva Anestesiologica*.

[26] Weil B. R., Herrmann J. L., Abarbanell A. M., Manukyan M. C., Poynter J. A., Meldrum D. R. (2011). Intravenous infusion of mesenchymal stem cells is associated with improved myocardial function during endotoxemia. *Shock*.

[27] Rogers T. B., Pati S., Gaa S. (2011). Mesenchymal stem cells stimulate protective genetic reprogramming of injured cardiac ventricular myocytes. *Journal of Molecular and Cellular Cardiology*.

[28] Rittirsch D., Flierl M. A., Ward P. A. (2008). Harmful molecular mechanisms in sepsis. *Nature Reviews Immunology*.

[29] Weil B. R., Manukyan M. C., Herrmann J. L. (2010). Mesenchymal stem cells attenuate myocardial functional depression and reduce systemic and myocardial inflammation during endotoxemia. *Surgery*.

[30] Chang C. L., Leu S., Sung H. C. (2012). Impact of apoptotic adipose-derived mesenchymal stem cells on attenuating organ damage and reducing mortality in rat sepsis syndrome induced by cecal puncture and ligation. *Journal of Translational Medicine*.

[31] Yip H. K., Chang Y. C., Wallace C. G. (2013). Melatonin treatment improves adipose-derived mesenchymal stem cell therapy for acute lung ischemia-reperfusion injury. *Journal of Pineal Research*.

[32] Strowig T., Henao-Mejia J., Elinav E., Flavell R. (2012). Inflammasomes in health and disease. *Nature*.

[33] Zhou R., Yazdi A. S., Menu P., Tschopp J. (2011). A role for mitochondria in NLRP3 inflammasome activation. *Nature*.

[34] Murakami T., Ockinger J., Yu J. (2012). Critical role for calcium mobilization in activation of the NLRP3 inflammasome. *Proceedings of the National Academy of Sciences of the United States of America*.

[35] Wu Y., Ren J., Zhou B. (2015). Gene silencing of non-obese diabetic receptor family (NLRP3) protects against the sepsis-induced hyper-bile acidaemia in a rat model. *Clinical and Experimental Immunology*.

[36] Ganz M., Csak T., Nath B., Szabo G. (2011). Lipopolysaccharide induces and activates the Nalp3 inflammasome in the liver. *World Journal of Gastroenterology*.

[37] Heid M. E., Keyel P. A., Kamga C., Shiva S., Watkins S. C., Salter R. D. (2013). Mitochondrial reactive oxygen species induces NLRP3-dependent lysosomal damage and inflammasome activation. *The Journal of Immunology*.

[38] Kim I., Rodriguez-Enriquez S., Lemasters J. J. (2007). Selective degradation of mitochondria by mitophagy. *Archives of Biochemistry and Biophysics*.

[39] Mahrouf-Yorgov M., Augeul L., Da Silva C. C. (2017). Mesenchymal stem cells sense mitochondria released from damaged cells as danger signals to activate their rescue properties. *Cell Death and Differentiation*.

[40] Hsieh C. H., Pai P. Y., Hsueh H. W., Yuan S. S., Hsieh Y. C. (2011). Complete induction of autophagy is essential for cardioprotection in sepsis. *Annals of Surgery*.

[41] Yen Y. T., Yang H. R., Lo H. C. (2013). Enhancing autophagy with activated protein C and rapamycin protects against sepsis-induced acute lung injury. *Surgery*.

[42] Lin C. W., Lo S., Perng D. S. (2014). Complete activation of autophagic process attenuates liver injury and improves survival in septic mice. *Shock*.

[43] Carchman E., Zuckerbraun B. (2011). Mitophagy/mitochondrial biogenesis is necessary to prevent organ injury in sepsis and is dependent on TLR9 signaling. *Journal of the American College of Surgeons*.

[44] Chan N. C., Salazar A. M., Pham A. H. (2011). Broad activation of the ubiquitin-proteasome system by Parkin is critical for mitophagy. *Human Molecular Genetics*.

