# Supplementary material for: A Novel Mechanism of Mesenchymal Stromal Cell-Mediated Protection against Sepsis: Restricting Inflammasome Activation in Macrophages by Increasing Mitophagy and Decreasing Mitochondrial ROS
Source: Oxid Med Cell Longev. 2018 Feb 13;2018:3537609. doi: 10.1155/2018/3537609 (PMC5831900; doi:10.1155/2018/3537609)
Supplement: Supplementary 1 — Figure S1: characterization and fate of injected of BMSCseGFP+. A. Differentiation potential of BMSCseGFP+. Fibroblast-like-shaped BMSCseGFP+ were GFP positive. Adipogenesis of BMSCseGFP+ under the adipogenic differentiation conditions was detected by oil red O staining. Osteogenesis was evaluated by Alizarin red S staining. Scale bar, 100 μm. B. Flow cytometry results show that BMSCseGFP+ were uniformly negative for CD34, CD45, and Sca-1 and positive for CD29, CD44, and CD90. C. Visualization of BMSCseGFP+ (red arrows) in C57BL/6 mice liver tissue at 1–6 h after intravenous injection. Scale bar, 100 μm. Data shown are representative of two (A, B) or three (C) independent experiments. [file 3537609.f1.pptx]

## Slide 1
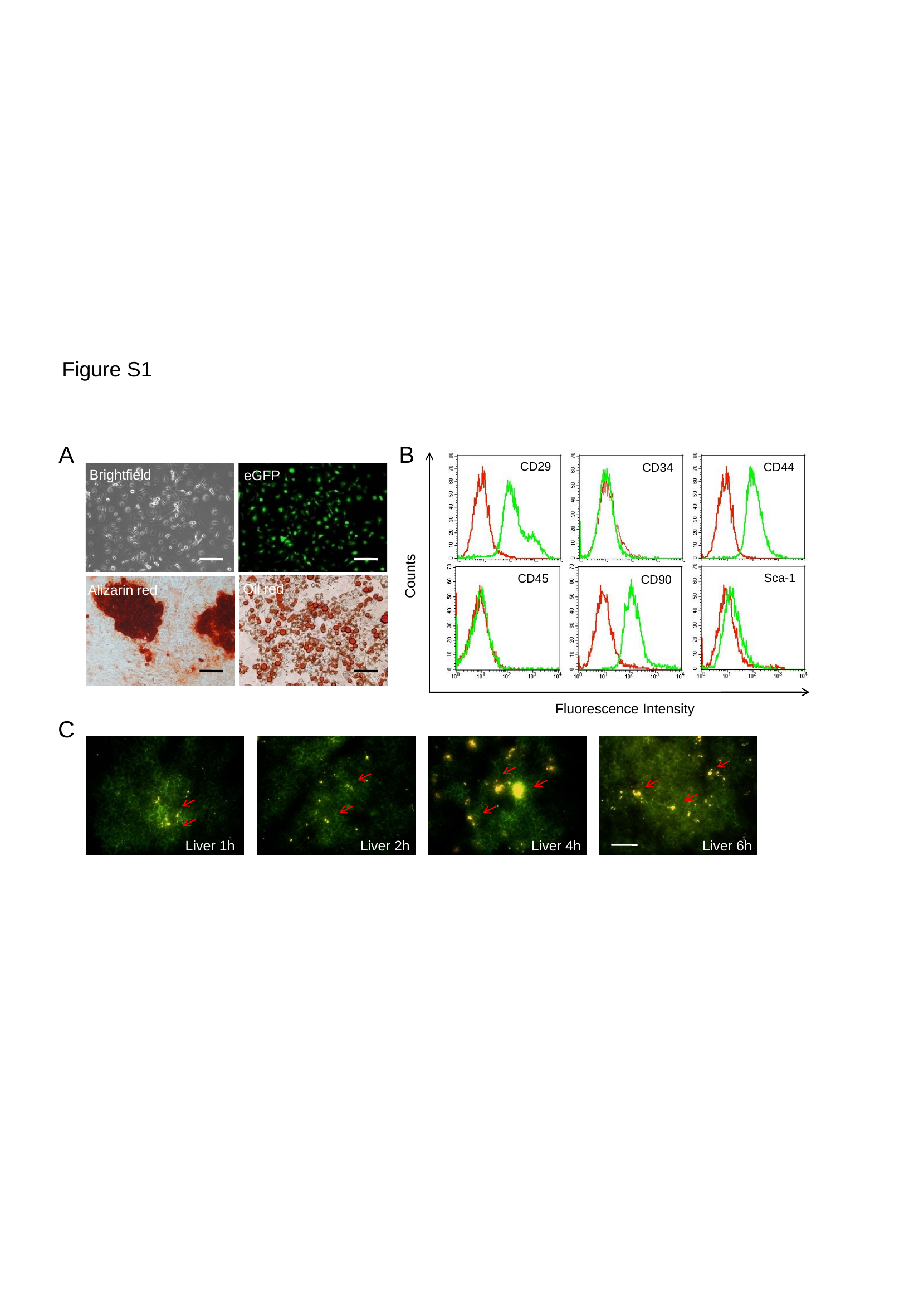

Figure S1
A
B
CD34
CD29
CD44
Brightfield
eGFP
Counts
Sca-1
CD45
CD90
Oil red
Alizarin red
Fluorescence Intensity
C
Liver 1h
Liver 2h
Liver 4h
Liver 6h
