# Supplementary material for: A Novel Mechanism of Mesenchymal Stromal Cell-Mediated Protection against Sepsis: Restricting Inflammasome Activation in Macrophages by Increasing Mitophagy and Decreasing Mitochondrial ROS
Source: Oxid Med Cell Longev. 2018 Feb 13;2018:3537609. doi: 10.1155/2018/3537609 (PMC5831900; doi:10.1155/2018/3537609)
Supplement: Supplementary 2 — Figure S2: inhibition of ROS generation abolished caspase-1 activation. A. Western blot analysis for caspase-1 and IL-1β in lysates of BMDMs incubated for 1 h with NAC (Sigma-Aldrich, USA) (10 mM), which was followed by LPS and ATP. B. Semiquantitative analysis of the Western blots. Error bars represent the means ± s.e.m. ∗ p < 0.05 and ∗∗ p < 0.01. n = 5–10. The data are representative of at least three independent experiments. [file 3537609.f2.pptx]

## Slide 1
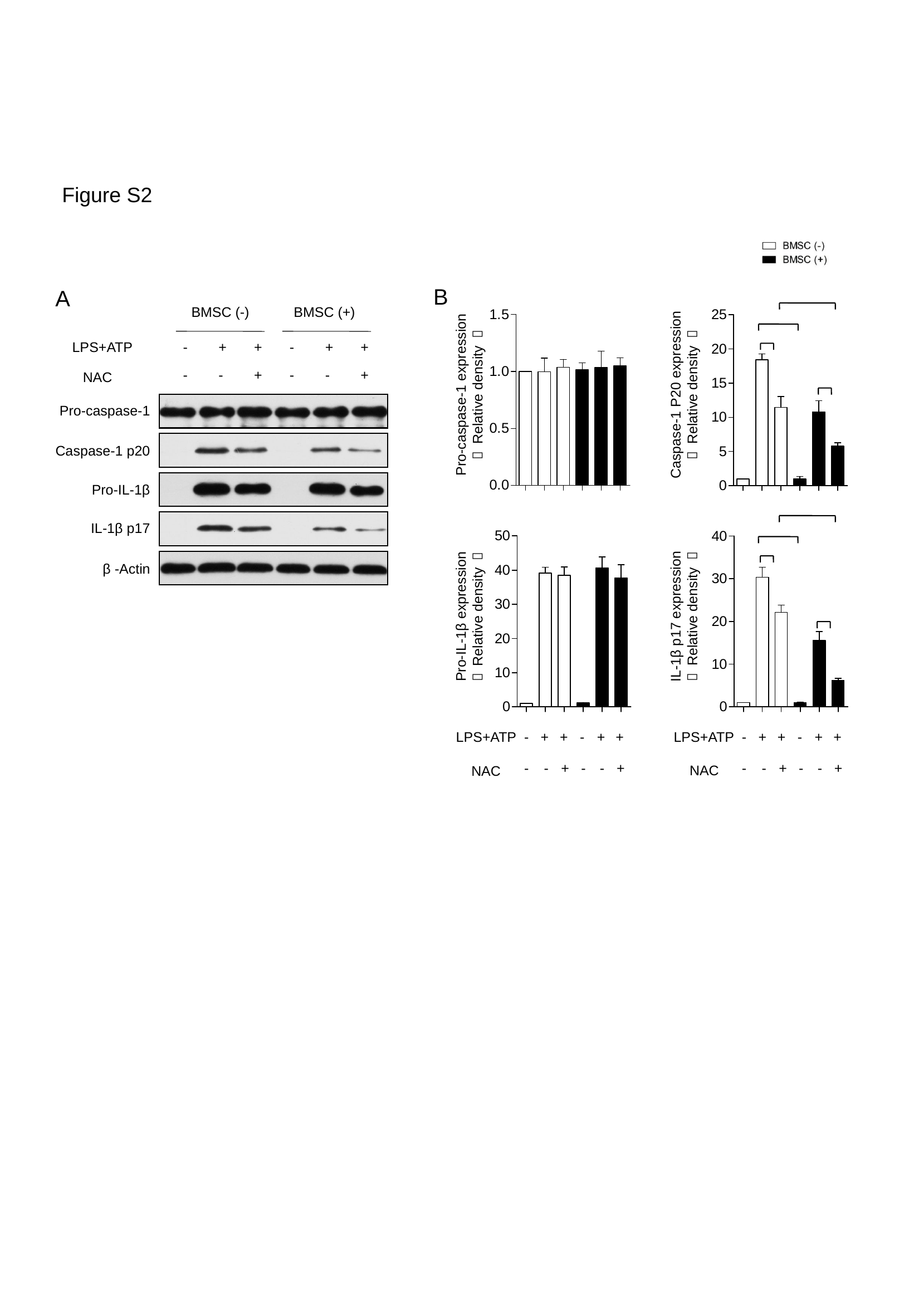

Figure S2
B
A
*
BMSC (-)
BMSC (+)
*
LPS+ATP
-
+
+
-
+
+
**
-
-
+
-
-
+
NAC
Caspase-1 P20 expression
（ Relative density ）
Pro-caspase-1 expression
（ Relative density ）
**
Pro-caspase-1
Caspase-1 p20
Pro-IL-1β
**
IL-1β p17
**
*
β -Actin
IL-1β p17 expression
（ Relative density ）
Pro-IL-1β expression
（ Relative density ）
**
LPS+ATP
-
+
+
-
+
+
-
-
+
-
-
+
NAC
LPS+ATP
-
+
+
-
+
+
-
-
+
-
-
+
NAC
