# Supplementary figures and images for: A Novel Mechanism of Mesenchymal Stromal Cell-Mediated Protection against Sepsis: Restricting Inflammasome Activation in Macrophages by Increasing Mitophagy and Decreasing Mitochondrial ROS
Source: Oxid Med Cell Longev. 2018 Feb 13;2018:3537609. doi: 10.1155/2018/3537609 (PMC5831900; doi:10.1155/2018/3537609)

## Slide 1
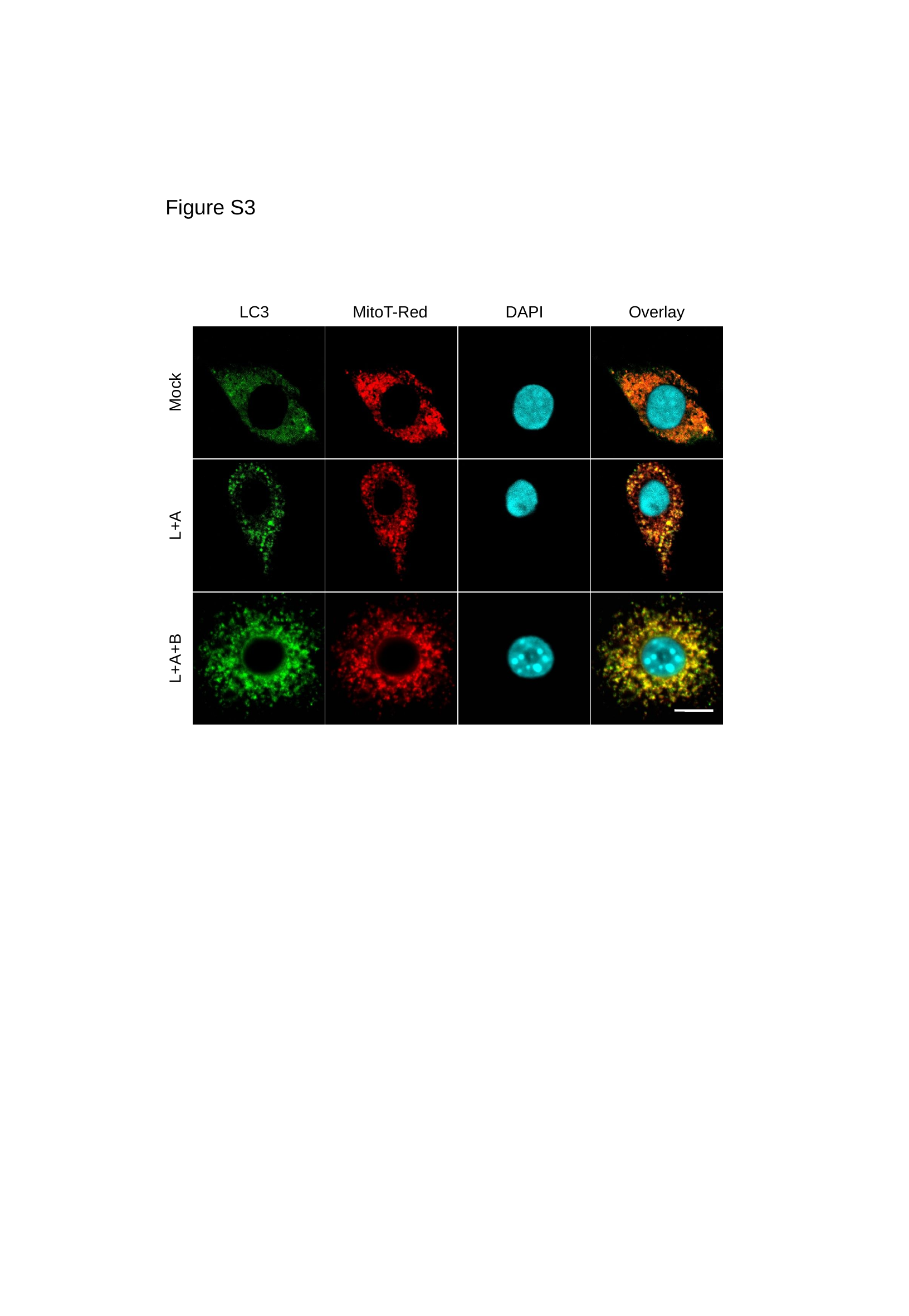

Figure S3
LC3
MitoT-Red
DAPI
Overlay
Mock
L+A
L+A+B

Supplement: Supplementary 3 — Figure S3: confocal microscopy analysis of BMDMs stained for LC3 (green) and mitochondria (red) for colocalization as an indicator of mitophagy. Scale bar, 20 μm. [file 3537609.f3.pptx]

## Slide 1
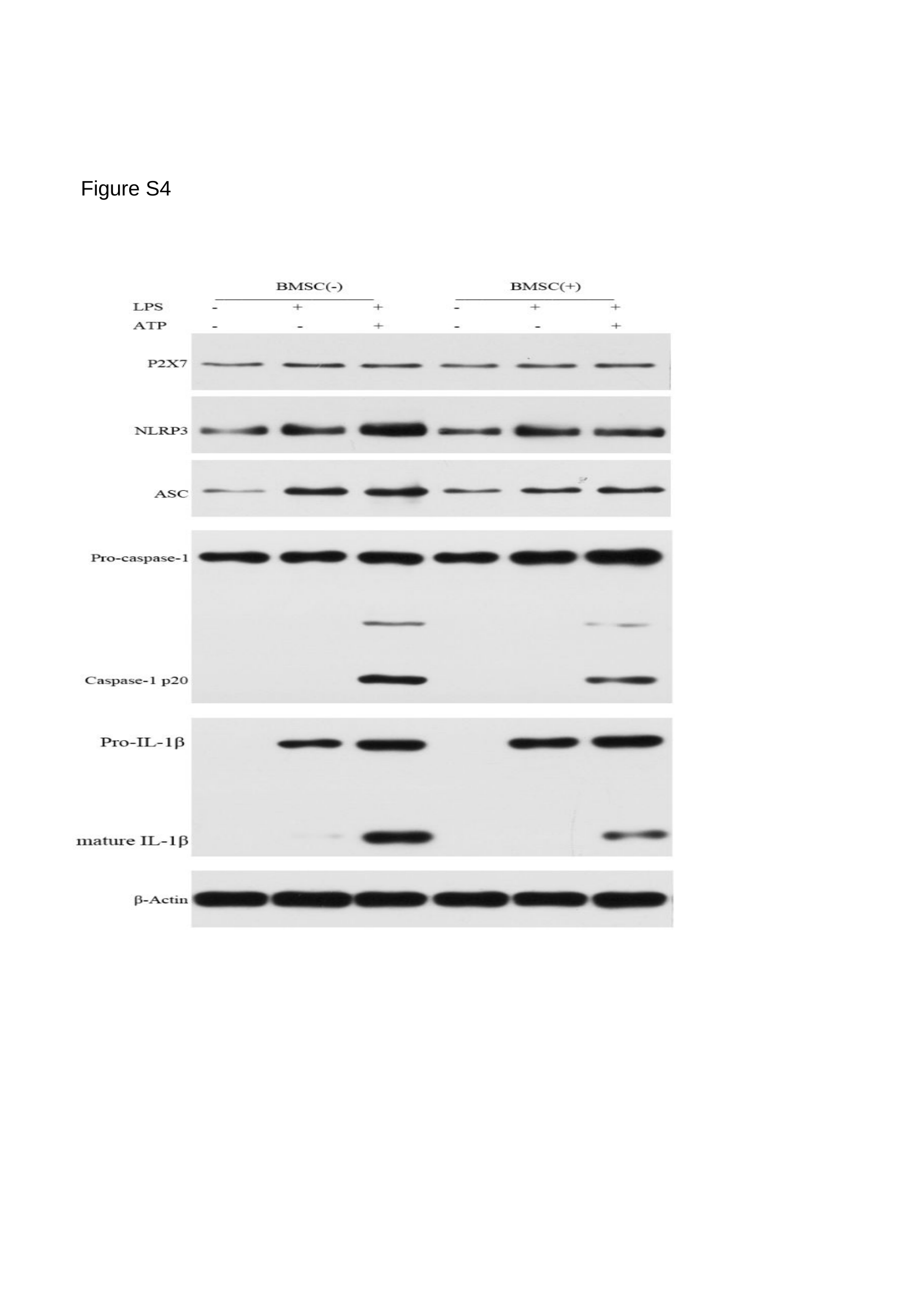

Figure S4

Supplement: Supplementary 4 — Figure S4: original images of caspase-1 p20 and IL-1β p17 in Figure 4. [file 3537609.f4.pptx]

## Slide 1
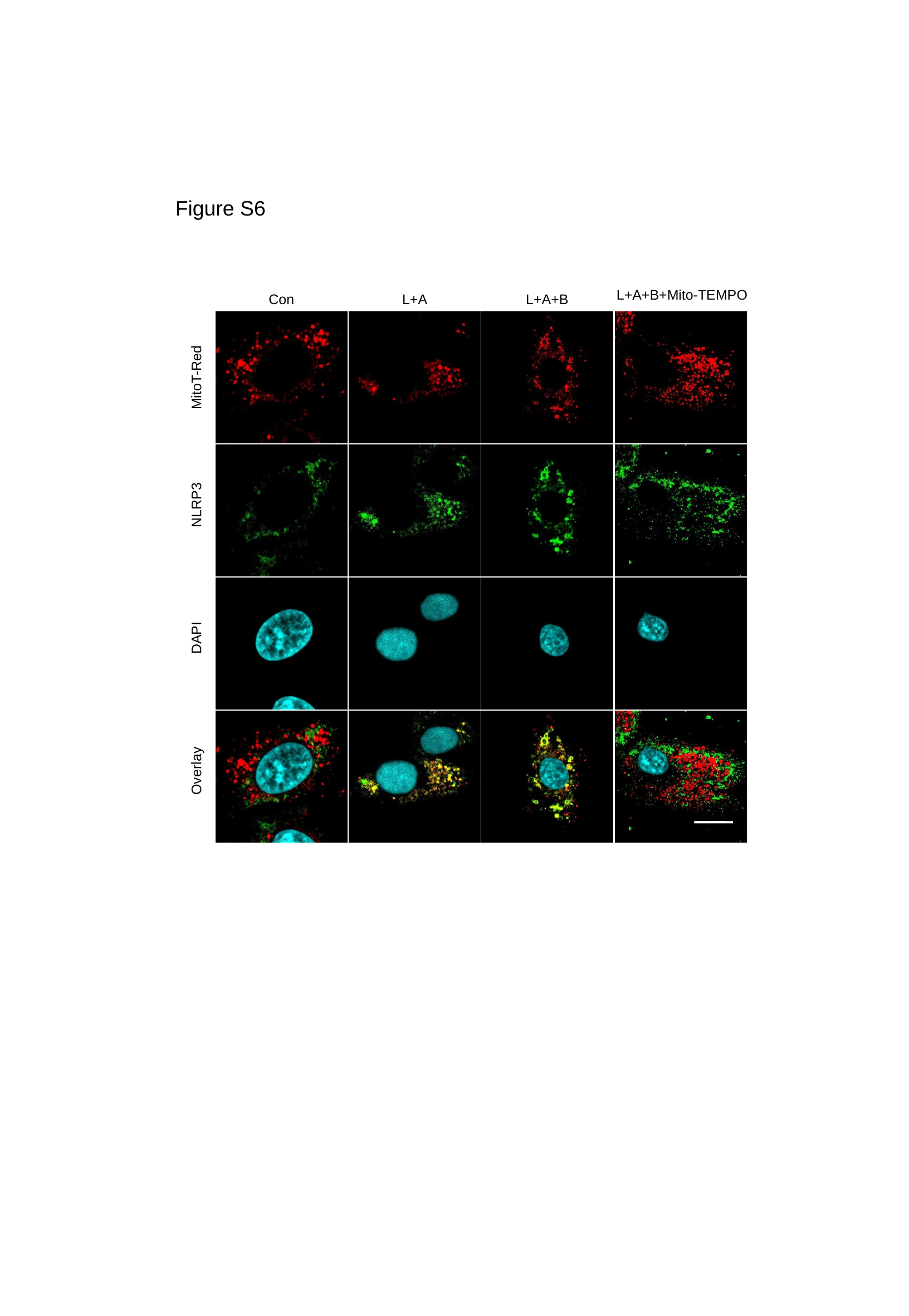

Figure S6
L+A+B+Mito-TEMPO
L+A+B
Con
L+A
MitoT-Red
NLRP3
DAPI
Overlay

Supplement: Supplementary 6 — Figure S6: colocalization of the NLRP3 and mitochondria. BMDMs expressing NLRP3 (green) were analyzed for the colocalization of NLRP3 with the mitochondria (red) using confocal microscopy. Scale bar, 20 μm. [file 3537609.f6.pptx]
